# Strategies to optimize MEDLINE and EMBASE search strategies for anesthesiology systematic reviews. An experimental study

**DOI:** 10.1590/1516-3180.2017.0277100917

**Published:** 2018-01-09

**Authors:** Enilze de Souza Nogueira Volpato, Marluci Betini, Maria Eduarda Puga, Arnav Agarwal, Antônio José Maria Cataneo, Luciane Dias de Oliveira, Rodrigo Bazan, Leandro Gobbo Braz, José Eduardo Guimarães Pereira, Regina El Dib

**Affiliations:** I PhD. Doctoral Student, Postgraduate Program on Anesthesiology, Health Sciences Library, Faculdade de Medicina de Botucatu (FMB), Universidade Estadual Paulista (UNESP), Botucatu (SP), Brazil.; II PhD. Coordinator, Coordenadoria da Rede de Bibliotecas da UNIFESP (CRBU), Universidade Federal de São Paulo (UNIFESP), São Paulo (SP), Brazil.; III Undergraduate Medical Student, School of Medicine, University of Toronto, Toronto, Ontario, Canada, and Department of Health Research Methods, Evidence and Impact, McMaster University, Hamilton, Ontario, Canada.; IV MD, PhD. Full Professor, Department of Surgery and Orthopedics, Faculdade de Medicina de Botucatu, Universidade Estadual Paulista (UNESP), Botucatu (SP), Brazil.; V MSc, PhD. Associate Professor, Department of Biosciences and Oral Diagnosis, Institute of Science and Technology, Universidade Estadual Paulista (UNESP), São José dos Campos (SP), Brazil.; VI MD. Assistant Professor, Department of Neurology, Faculdade de Medicina de Botucatu, Universidade Estadual Paulista (UNESP), Botucatu (SP), Brazil.; VII MD. Assistant Professor, Department of Anesthesiology, Faculdade de Medicina de Botucatu, Universidade Estadual Paulista (UNESP), Botucatu (SP), Brazil.; VIII MD. Doctoral Student, Postgraduate Program on Anesthesiology, Faculdade de Medicina de Botucatu, Universidade Estadual Paulista (UNESP), Botucatu (SP), Brazil.; IX MSc, PhD. Assistant Professor, Department of Anesthesiology, Faculdade de Medicina de Botucatu, Universidade Estadual Paulista (UNESP), Botucatu (SP), Brazil; Assistant Professor, Department of Biosciences and Oral Diagnosis, Institute of Science and Technology, Universidade Estadual Paulista (UNESP), São José dos Campos (SP), Brazil; and Research Collaborator, Institute of Urology, McMaster University, Hamilton, Ontario, Canada.

**Keywords:** Evidence-based medicine, MEDLINE, Databases, bibliographic, Medical subject headings, Anesthesiology

## Abstract

**BACKGROUND:**

A high-quality electronic search is essential for ensuring accuracy and comprehensiveness among the records retrieved when conducting systematic reviews. Therefore, we aimed to identify the most efficient method for searching in both MEDLINE (through PubMed) and EMBASE, covering search terms with variant spellings, direct and indirect orders, and associations with MeSH and EMTREE terms (or lack thereof).

**DESIGN AND SETTING:**

Experimental study. UNESP, Brazil.

**METHODS:**

We selected and analyzed 37 search strategies that had specifically been developed for the field of anesthesiology. These search strategies were adapted in order to cover all potentially relevant search terms, with regard to variant spellings and direct and indirect orders, in the most efficient manner.

**RESULTS:**

When the strategies included variant spellings and direct and indirect orders, these adapted versions of the search strategies selected retrieved the same number of search results in MEDLINE (mean of 61.3%) and a higher number in EMBASE (mean of 63.9%) in the sample analyzed. The numbers of results retrieved through the searches analyzed here were not identical with and without associated use of MeSH and EMTREE terms. However, association of these terms from both controlled vocabularies retrieved a larger number of records than did the use of either one of them.

**CONCLUSIONS:**

In view of these results, we recommend that the search terms used should include both preferred and non-preferred terms (i.e. variant spellings and direct/indirect order of the same term) and associated MeSH and EMTREE terms, in order to develop highly-sensitive search strategies for systematic reviews.

## INTRODUCTION 

A high-quality electronic search is essential for ensuring accuracy and comprehensiveness among the records retrieved when conducting systematic reviews.^1^ The quality of the records retrieved depends on the way in which sensitive search strategies are formulated and on the use of appropriate electronic and non-electronic databases.^2^ To achieve such quality, the researchers need to be familiar with the controlled retrieval languages and the tools available in each database.^3^

With the introduction and dissemination of evidence-based medicine within anesthesiology, there has been a growing focus on evaluation of the coverage, scope and limitations of databases^4^^,^^5^^,^^6^ and search strategies^2^^,^^7^^,^^8^^,^^9^ for anesthesiology-related systematic reviews.

For the purposes of indexing and searching, sets of equivalent terms are generally treated as having the same meaning, and as such, are represented by a single preferred term.^10^ Non-preferred terms include variant spellings (e.g. closed-circuit anesthesia versus closed circuit anesthesia), direct and indirect ordering (e.g. anesthesia, rebreathing, versus rebreathing anesthesia) and synonyms (e.g. neoplasm and cancer). It is important to take into account both preferred and non-preferred terms in developing sensitive search strategies. 

Furthermore, it is important to select all search terms that may represent the subject under investigation, for inclusion in the search strategy. Inclusion of both database subject headings and text words retrieves papers that would not have been found if only the subject headings had been searched for.^11^ Terms can be selected based on terms used by the authors of the article, or on keywords, or by consulting a controlled vocabulary or thesaurus (i.e. MeSH and EMTREE terms for the MEDLINE and EMBASE databases, respectively).

To assist researchers in identifying appropriate terms for a sensitive search strategy, librarians and educators recommend consulting and including preferred and non-preferred terms from a controlled database vocabulary.^11^ However, by using all available terms in the thesaurus (i.e. subject headings), strategy development may be lengthy and very laborious. One additional factor influencing the choice of terms within anesthesiology is the users’ own practical clinical experience.

## OBJECTIVE

In this paper, we explored the use of preferred and non-preferred search terms and MeSH/EMTREE terms in the MEDLINE and EMBASE search strategies for systematic reviews with anesthesiology. The purposes of this study were to ascertain:

1. whether variant spellings and inclusion of direct and indirect ordering of the same terms retrieved the same number of records; and
2. whether inclusion of appropriate MeSH and EMTREE terms retrieved a larger number of records than would use of either MeSH alone or EMTREE alone.

## METHODS

In our experimental study, we selected 37 terms in the field of anesthesiology from the MeSH and EMTREE databases, and then we analyzed 37 search strategies that were derived from those terms. These search strategies were adapted to include search terms with variant spellings, direct and indirect ordering, and related MeSH and EMTREE terms. The adapted searches were re-run in the MEDLINE (via PubMed) and EMBASE databases (Table 1). We did not impose any year restrictions. The databases were searched starting from their inception: Elsevier MEDLINE via PubMed from 1946 to January 2017; and Elsevier EMBASE from 1947 to January 2017. The cutoff date was January 15, 2017.

**Table 1:** Comparison of results retrieved through the 37 search strategies, either with or without use of variations in the MEDLINE (via PubMed) and EMBASE databases

Comparison between with and without variations		Equal numbers of results retrieved (%)	Greater number of results retrieved with variations (%)	Smaller number of results retrieved with variations (%)	P-value
MEDLINE via PubMed	MeSH	27 (73.0)^a^	10 (27.0)^b^	0 (0.0)^c^	P < 0.0001
	MeSH + EMTREE	20 (54.0)^a^	16 (43.3)^a^	1 (2.7)^b^	P < 0.0001
	EMTREE	21 (56.8)^a^	14 (37.8)^a^	2 (5.4)^b^	P < 0.0001
EMBASE	MeSH	12 (32.4)^a^	25 (67.6)^b^	0 (0.0)^c^	P < 0.0001
	MeSH + EMTREE	10 (27.0)^a^	26 (70.3)^b^	1 (2.7)^c^	P < 0.0001
	EMTREE	16 (43.3)^a^	20 (54.0)^a^	1 (2.7)^b^	P < 0.0001

a, b, c = values followed by the same letter did not differ significantly.

We chose simple terms in the field of anesthesiology through discussion with our co-authors with expertise in anesthesiology. These terms were searched for in the MeSH database, from which 237 potential subject headings were retrieved. From these, we selected 37 MeSH terms that met the inclusion criteria, after removal of duplicates. Therefore, if a MeSH term presented only one of the main two criteria described above, we excluded it.

In adapting the search strategies, the so-called preferred and non-preferred terms were identified based on the following search term eligibility criteria:

1. variant spellings,
  1. with or without a hyphen (e.g. closed-circuit anesthesia versus closed circuit anesthesia);
  2. with or without a space (e.g. anti-inflammatory agents, non-steroidal versus antiinflammatory agents, nonsteroidal);
  3. American or British English (e.g. inhalation anesthesia versus inhalation anaesthesia); and
2. direct or indirect order (e.g. anesthesia, rebreathing, versus rebreathing anesthesia). For simplicity, the use of variant spellings for a given term is hereafter referred to as “with variations”, and the use of only one spelling for a given term is referred to as “without variations”. EMTREE terms were selected based on the corresponding preferred term for a MeSH term.

The original and adapted search strategies were run on the same day to avoid differences in the number of indexed records in the databases searched. The searches in MEDLINE and EMBASE were conducted using a consistent approach, with preservation of default configurations for both indexes, and without any application of language, period, type of study or other filters.

### Sample size

To estimate the sample size, we assumed that, across all the search strategies analyzed, 95% of the adapted search strategies with different models would show the same number of retrieved references. An error rate of 7% within a 95% confidence interval was assumed. Based on these assumptions, it was necessary to analyze approximately 37 search strategies, according to the following equation:

E = Z√pq/n

Where E is the sample error (0.07); Z is a constant relative to a 95% confidence interval (1.96); p corresponds to the expected proportion of records retrieved; and q is the complement of p regarding the total number of systematic reviews (1 - P).

### Statistical analysis 

We used Kruskal-Wallis ranked analysis of variance and the SAS software (SAS 9.3 Help and Documentation, SAS Institute Inc., Cary, NC, USA) for the statistical analysis. We expressed the number of searches as absolute numbers and percentages. We considered P*-*values of less than 0.05 to be statistically significant.

## RESULTS

The numbers of results retrieved across all 37 sets of search strategies are shown in Tables 1, 2, 3, 4 and 5.

**Table 2:** Comparison of results retrieved through the 37 search strategies, considering use of MeSH and EMTREE terms separately or in association, with variations, in the MEDLINE database (via PubMed)

Use of MeSH and EMTREE terms separately or in association, with variations		Equal numbers of results retrieved (%)	Greater number of results retrieved with the first variable^**†**^ (%)	Smaller number of results retrieved with the first variable^**†**^ (%)	P-value
MEDLINE via PubMed	MeSH^†^ versus MeSH + EMTREE	3 (8.1)^a^	0 (0.0)^b^	34 (91.9)^b^	P < 0.0001
	EMTREE^†^ versus MeSH + EMTREE	15 (40.6)^a^	2 (5.4)^b^	20 (54.0)^a^	P < 0.0001
	MeSH^†^ versus EMTREE	0 (0.0)^a^	12 (32.4)^b^	25 (67.6)^c^	P < 0.0001

^†^First variable is the one that is quoted first in the comparison of interest. a, b, c = values followed by the same letter did not differ significantly.

**Table 3:** Comparison of results retrieved through the 37 search strategies, considering use of MeSH and EMTREE terms separately or in association, without variations, in the MEDLINE database (via PubMed)

Use of MeSH and EMTREE terms separately or in association, without variations		Equal numbers of results retrieved (%)	Greater number of results retrieved with the first variable^**†**^ (%)	Smaller number of results retrieved with the first variable^**†**^ (%)	P-value
MEDLINE via PubMed	MeSH^†^ versus MeSH + EMTREE	6 (16.2)^a^	0 (0)^b^	31 (83.8)^c^	P < 0.0001
	EMTREE^†^ versus MeSH + EMTREE	12 (32.4)^a^	4 (10.8)^b^	21 (56.8)^a^	P = 0.0010
	MeSH^†^ versus EMTREE	1 (2.7)^a^	12 (32.4)^b^	24 (64.9)^c^	P < 0.0001

^†^First variable is the one that is quoted first in the comparison of interest. a, b, c = values followed by the same letter did not differ significantly.

**Table 4:** Comparison of results retrieved through the 37 search strategies, considering use of MeSH and EMTREE terms separately or in association, with variations, in the EMBASE database

Use of MeSH and EMTREE terms separately or in association, with variations		Equal numbers of results retrieved (%)	Greater number of results retrieved with the first variable^**†**^ (%)	Smaller number of results retrieved with the first variable^**†**^ (%)	P-value
EMBASE	MeSH^†^ versus MeSH + EMTREE	7 (18.9)^a^	1 (2.7)^b^	29 (78.4)^c^	P < 0.0001
	EMTREE^†^ versus MeSH + EMTREE	13 (35.1)^a^	0 (0)^b^	24 (64.9)^c^	P < 0.0001
	MeSH^†^ versus EMTREE	2 (5.40)^a^	13 (35.1)^b^	22 (59.5)^c^	P < 0.0001

^†^First variable is the one that is quoted first in the comparison of interest. a, b, c = values followed by the same letter did not differ significantly.

**Table 5:** Comparison of results retrieved through the 37 search strategies, considering use of MeSH and EMTREE terms separately or in association, without variations, in the EMBASE database

Use of MeSH and EMTREE terms separately or in association, without variations		Equal numbers of results retrieved (%)	Greater number of results retrieved with the first variable^**†**^ (%)	Smaller number of results retrieved with the first variable^**†**^ (%)	P-value
EMBASE	MeSH^†^ versus MeSH + EMTREE	9 (24.3)^a^	1 (2.7)^b^	27 (73.0)^c^	P < 0.0001
	EMTREE^†^ versus MeSH + EMTREE	15 (40.6)^a^	4 (10.8)^b^	18 (48.6)^a^	P = 0.0045
	MeSH^†^ versus EMTREE	4 (10.8)^a^	12 (32.4)^b^	21 (56.8)^c^	P < 0.0001

^†^First variable is the one that is quoted first in the comparison of interest. a, b, c = values followed by the same letter did not differ significantly.

In the MEDLINE via PubMed database, in comparing search strategies with variant spellings, the majority of the search strategies retrieved the same number of records through the three different approaches: 73.0% in the strategies only using MeSH terms; 54.0% using MeSH and associated EMTREE terms; and 56.8% only using EMTREE terms (P < 0.0001) (Table 1). With regard to EMBASE, the searches with variations recovered more records than the ones without variations: only using MeSH terms, 67.7%; using the association of MeSH with EMTREE terms, 70.3%; and only using EMTREE terms, 54.0% (P < 0.0001) (Table 1).

Among the search strategies conducted in MEDLINE through PubMed with variations, the majority retrieved a smaller number of results through only using MeSH, compared with using MeSH and EMTREE together (91.9%); only using EMTREE, compared with using MeSH and EMTREE together (54.0%); and only using MeSH compared with only using EMTREE (67.6%) (P < 0.0001) (Table 2). Similar results were found using search strategies without variations, through comparing only using MeSH (83.8%) and only using EMTREE (56.8%) with using MeSH and EMTREE together; and through comparing only using MeSH with only using EMTREE terms (64.9%) (P < 0.0001 for all comparisons) (Table 3).

In EMBASE, search strategies involving associated MeSH and EMTREE terms identified more records than those only using MeSH terms or only using EMTREE terms, regardless of term variation (P < 0.0001) (Tables 4 and 5).

## DISCUSSION

There are already many articles that explain the rules for searches in the literature. However, these are related to the use of filters rather than the construction of the search strategy itself. Furthermore, there are very few studies testing models for search strategies applicable to systematic reviews. The search strategy model that we used in this study is found in clinical practice among scientific investigators who wish to perform systematic reviews. However, this model had never been scrutinized through the rigor of scientific methodology.

Therefore, in this study, we compared the numbers of records with inclusion of variant spellings and inclusion of direct and indirect ordering, by means of three different formulations (i.e. MeSH, MeSH + EMTREE, and EMTREE) using identical search strategies. In other words, the same keywords and Boolean operators were used to test variant spellings, direct and indirect orders and associations of MeSH and EMTREE terms (or lack thereof) in MEDLINE via PubMed and in EMBASE, to identify the best approaches towards formulating search strategies for systematic reviews within anesthesiology. In this study, we did not aim to analyze the relevance of the papers retrieved (i.e. specificity).

Among the 37 search strategies run in MEDLINE via PubMed, 10 formulated only using MeSH terms retrieved fewer articles when the search was done without variations. In EMBASE, 20 search strategies formulated only using EMTREE terms retrieved more records when they were run with variations than without variations. While it may be ideal from a feasibility and efficiency perspective to conduct searches without variations, accounting for these variations appears integral to the formulation of a sensitive search strategy.

No variables were identified as being clearly predictive of search strategies in which inclusion of variations might be more beneficial in terms of the numbers of records identified. We initially hypothesized that searches using preferred terms that presented a higher number of non-preferred terms might be associated with differences in numbers of records identified when searched for with or without variations. However, both the searches formulated using the term “headache”, which presented the greatest number of non-preferred terms (57 terms), and the searches using the term “delayed emergence from anesthesia” (55 non-preferred terms) retrieved the same number of records in searches conducted with and without variations, in MEDLINE with the use of MeSH terms alone.

We also considered whether the type of variation could have interfered with the results. However, no such association was found between the type of variation and the number of records identified.

The number of search strategies formulated only using MeSH terms in MEDLINE that retrieved the same number of results with or without variations was greater than the number of strategies formulated only using EMTREE terms in EMBASE that did the same. This may indicate that the controlled vocabulary of MeSH might be more structured, while EMTREE terms are more comprehensive.

In many published systematic reviews, we noticed that MeSH terms alone were often used in the search strategies. However, after we ran the searches only using MeSH, using MeSH plus EMTREE and only using EMTREE, we found that the numbers of results retrieved through the searches analyzed were greater using MeSH plus EMTREE in MEDLINE than using the same association in EMBASE. Considering that the EMBASE index system has greater depth than MEDLINE,^12^ especially in relation to the field of pharmacology, in which most of our terms were classified (45%), use of an association of both MeSH and EMTREE terms in EMBASE has a lower impact than does use of the same association in the MEDLINE database. Therefore, if researchers want to find the maximum number of results through a search strategy for a particular topic, they should use both MeSH and EMTREE.

Our study has several limitations that should be considered. Firstly, while 37 studies provided us with an adequate sample size based on sample size calculations, a larger systematic analysis on search strategies might provide findings of greater robustness. Secondly, while the number of hits identified is one means of measuring the comprehensiveness of search strategies, no effort was made to examine the records identified regarding their relevance to the given research question. It is possible that certain search strategies identified more records but were less focused on the research question, or missed eligible studies that were identified through other search strategies. Thirdly, our analysis was limited to the MEDLINE and EMBASE electronic databases. To achieve a more comprehensive analysis, other electronic databases and other indexed sources commonly used in systematic review searches should be evaluated, to better inform effective search strategy formulation when using these sources.

There are very few studies evaluating different models for building searching strategies. A study^9^ with the aim of identifying the best method for searching in MEDLINE through PubMed, which considered whether parentheses, double quotation marks and truncation should be used or whether a simple search or search history should be used, found that there was no need to use phrase-searching parentheses to retrieve studies. However, the authors of that study recommended the use of double quotation marks when an investigator was attempting to retrieve articles in which a term appeared to be exactly the same as what was proposed in the search form.

Given that systematic reviews use rigorous methods to identify, critically appraise and synthesize relevant research studies, we also need to be aware of the best tools for implementing comprehensive search strategies, depending on the clinical question, in order to ensure that the results will be as current as possible and not be biased. Identifying optimal strategies for developing comprehensive and sensitive search strategies is fundamental to conducting rigorous systematic reviews.^13^

Our study found that the number of records retrieved in the MEDLINE (via PubMed) and EMBASE databases when search strategies were formulated using MeSH and/or EMTREE terms with variations (including variant spellings and direct and indirect ordering) differed from the number retrieved through the same strategies without these variations. Furthermore, using associated MeSH and EMTREE terms when conducting searches in both the MEDLINE (via PubMed) and EMBASE databases identified a greater number of records than did using only the EMTREE terms or only the MeSH terms.

## CONCLUSIONS

In view of these results, we recommend inclusion of all preferred and non-preferred terms (variant spellings and direct/indirect orders of terms), and associated MeSH and EMTREE terms, when searching the MEDLINE (via PubMed) and EMBASE databases, in formulating sensitive search strategies for systematic reviews.
