# ERRATUM

**DOI:** 10.1590/1516-3180.2017.0277100917erratum

**Published:** 2018-03-05

In the manuscript “Strategies to optimize MEDLINE and EMBASE search strategies for anesthesiology systematic reviews. An experimental study”, published in the Sao Paulo Med J. 2018 Jan 15:0. doi: 10.1590/1516-3180.2017.0277100917. [Epub ahead of print]:

Where it read:

“Volpato ESN, Betini M, Puga ME, Agarwal A, Cataneo AJM, Oliveira LD, Ferreira RP, Bazan R, Braz LG, Pereira JEG, Dib RE”

It should read:

“Volpato ESN, Betini M, Puga ME, Agarwal A, Cataneo AJM, Oliveira LD, Bazan R, Braz LG, Pereira JEG, El Dib R”

